# BMI trajectories and risk of overall and grade‐specific prostate cancer: An observational cohort study among men seen for prostatic conditions

**DOI:** 10.1002/cam4.1747

**Published:** 2018-09-11

**Authors:** Kai Wang, Xinguang Chen, Travis A. Gerke, Victoria Y. Bird, Hans K. Ghayee, Mattia Prosperi

**Affiliations:** ^1^ Department of Epidemiology University of Florida Gainesville Florida; ^2^ Department of Cancer Epidemiology Moffitt Cancer Center Tampa Florida; ^3^ Department of Urology University of Florida Gainesville Florida; ^4^ Department of Internal Medicine Division of Endocrinology University of Florida and the Malcom Randall VA Medical Center Gainesville Florida

**Keywords:** body mass index (BMI), developmental trajectory analysis, Gleason score, middle‐to‐late adulthood, prostate cancer

## Abstract

**Background:**

Dynamic longitudinal patterns in body mass index (BMI) have been suggested to better predict health outcomes than static measures. Effects of BMI trajectories on prostate cancer (PCa) risk have not been thoroughly explored.

**Methods:**

Cohort data were derived from electronic medical records of patients who were admitted to a tertiary‐care hospital in the Southeastern USA during 1994‐2016. Patients with a history of urologic clinic visit because of any prostatic condition and with repeatedly measured BMI (n = 4857) were included. BMI trajectories prior to PCa diagnosis were assessed using the developmental trajectory analysis method. Cox proportional hazards regression modeling was used to estimate adjusted hazard ratio (aHR) with 95% confidence intervals (CIs) for overall and grade‐specific PCa.

**Results:**

The median age (interquartile range, IQR) of the participants at baseline was 63 (54, 72) years. Over a median follow‐up (IQR) of 8.0 (2.0, 13.0) years, 714 (14.7%, 714/4857) were diagnosed with PCa. Men with growing BMI trajectory progressing from normal weight to overweight/obese had a 76% increased PCa risk (aHR = 1.76; 95% CI: 1.25, 2.48), and men being obese and experiencing progressive weight gain had 3.72‐fold increased PCa risk (aHR = 3.72; 95% CI: 1.60, 8.66), compared to men with persistently normal BMI. The associations were more pronounced for PCa with Gleason score ≥7. No significant association of decreasing BMI trajectory progressing from obese to normal BMI was found with PCa risk.

**Conclusions:**

Progressively body weight gain during middle‐to‐late adulthood was associated with increased PCa risk for both normal weight and overweight men. Further studies are warranted to confirm this finding.

## 1 INTRODUCTION

The National Health and Nutrition Examination Survey (NHANES, 2013‐2014) found that the prevalence of obesity, defined as a body mass index (BMI) of 30 or more, has increased to 37.7% of all adults in the USA.^1^ BMI measures have been often positively associated with risk of a variety of cancers in adults, including esophageal, colorectal, pancreatic, and breast.^2^, ^3^, ^4^ However, findings regarding the relationship between BMI and risk of prostate cancer (PCa) are not conclusive.^5^, ^6^, ^7^, ^8^, ^9^ PCa is the leading cancer diagnosis among U.S. men. At the same token, the prevalence of obesity is expected to continue to increase in the coming decade.^10^ Thus, it is vital to improve understanding of the role of obesity, a modifiable factor in PCa etiology to optimize screening, prevention, and treatment.

Evidence from published studies appears to suggest a dual effect of high BMI on PCa risk: an increased risk of aggressive PCa and a decreased risk of localized PCa.^11^ However, most of these studies reported the association with static BMI measurements, that is, BMI measured at baseline, cumulative averages over time, or at most changes in BMI between two time‐points.^11^, ^12^, ^13^, ^14^, ^15^, ^16^ Evidence derived from these types of studies may be adequate to understand PCa risk with a static perspective, but obviously inadequate to capture the relevant etiologic window of PCa as the prostate carcinogenesis is a protracted course which can initiate as early as in the third decade of life.^17^, ^18^ Therefore, associating dynamic longitudinal patterns in BMI with PCa may better capture the true underlying effect of body weight change on the risk of incident PCa. However, a paucity of data persists in the literature regarding longitudinal BMI trajectories and PCa risk.

Two previous studies have examined BMI trajectories or body shape trajectories in relation to PCa risk.^19^, ^20^ Findings of these studies suggest that overweight males with no change in body weight or progressing from overweight to obese had a lower PCa risk, compared to men with persistently normal BMI. However, findings of these studies could be biased due to several issues. First, in these studies, self‐reported data were used together with recorded data to determine BMI trajectory. Second, BMI trajectories were determined using data measured at a limited number of time‐points, insufficient to characterize the potentially complex distinctive trajectories. Last, the conclusion that body weight gain is associated with reduced PCa risk is incongruent with the conclusions derived in studies targeting other cancers types.^2^, ^3^, ^4^

In the current study, we analyzed a longitudinal dataset with BMI calculated using body weight and height measured over a median period of 8 years in clinical settings. By a simultaneous examination of the baseline BMI and longitudinal BMI trajectories in relation to risk of overall PCa and PCa by Gleason grade, this study helps to disentangle the impact of BMI trajectories on PCa risk.

## 2 MATERIALS AND METHODS

### 2.1 Study population

We conducted a hospital‐based observational cohort study at a tertiary‐care hospital in the Southeastern USA. This study targeted patients with a history of urologic clinic visit because of any prostatic condition, including elevated prostate‐specific antigen (PSA) (>4 ng/mL). Firstly, International Classification of Disease, Ninth Revision (ICD‐9) codes for all prostatic conditions (Table S1) were used to screen for potential participants on the electronic medical records (EMR) system. We located patients with at least one of the codes shown in Table S1 at any hospital admission; then, medical records for all of his previous and subsequent hospital admissions (including outpatient visit and hospitalization) were extracted using an unique de‐identified patient ID. This ID was also used to link demographics, medical diagnoses, laboratory results, and drug prescription. We included patients that (a) were not PCa patient at the first hospital admission; (b) with repeated follow‐up assessments of height and weight after the first hospital admission. Only data measured before or at the time of PCa diagnosis or censoring (for non‐PCa patients) were analyzed. Patients not diagnosed with PCa were censored at date of the last hospital admission. To ensure internal validity, we excluded patients (a) diagnosed with PCa at the first hospital admission; or (b) diagnosed with PCa or censored before age 40, due to the potentially differing disease etiology in early‐onset PCa; or (c) with height and weight measured but only once or twice, inadequate for assessing BMI trajectories over time; or (d) with height and weight measured three or more times but not in three or more different calendar years, also inadequate for BMI trajectory analysis; or (e) being outliers with BMI<16.6 (<1.0 percentile) or >60.9 kg/m^2^ (>99.0 percentile). With these criteria, a total of 4857 participants were included in this analysis with a median follow‐up duration of 8.0 years over 39 078 person‐years exposure. For them, the first hospital admission was used as baseline, and subsequent hospital admissions were treated as follow‐ups. We accessed to the EMR database through the Informatics for Integrating Biology and the Bedside (i2b2) mechanism hosted by the Clinical and Translational Study Institute (CTSI) at the University of Florida. The included medical records ranged from 4 November 1994 to 10 January 2016.

This study has been performed in accordance with the Declaration of Helsinki. The research protocol has been approved by the University of Florida's Institutional Review Board.

### 2.2 Measurement of BMI

Height and weight were measured by healthcare providers at hospital admissions. We used data for height and weight up to the date when a PCa diagnosis was made. BMI was calculated as weight in kilograms divided by the square of height in meters (kg/m^2^). If a patient had more than one BMI measurement within one calendar year, the arithmetic mean was used to represent the BMI value for that year. BMI was analyzed as a continuous variable to determine the developmental trajectories over time. BMI was also categorized for analysis using the definition by the World Health Organization (WHO): underweight (<18.5), normal weight (18.5 to <25.0), overweight (25.0 to <30), class 1 obese (30.0 to <35.0) and class 2 or more obese (≥35.0).^21^ Since only 69 (1.4%) were underweight, these participants were combined with the normal weight group for analysis.

### 2.3 Determination of PCa and Gleason grade

The outcome of interest was newly diagnosed PCa. In addition to clinical symptoms and signs, PCa diagnosis was made based on evidence from PSA testing, result from digital rectal examination, ultrasound, multi‐parametric MRI, CT scan, supported by microscopic histopathologic characters of cancer tissue biopsy. The International Classification of Disease, Ninth Revision (ICD‐9) was used to determine a PCa diagnosis. Participant were coded as PCa cases if the medical record showing the ICD‐9 code = 185 (malignant neoplasm of prostate, equivalent to ICD‐10 CM C61) or 233.4 (in situ carcinoma prostate, equivalent to ICD‐10 CM D70.5). Of the total sample of 4857 participants, 714 (14.7%) were detected as new PCa cases during the follow‐up period.

For all PCa patients, Gleason scores were also derived from the electronic medical records. Using the Gleason score = 7 as the cutoff point, among the 714 PCa patients, 626 (87.7%) were classified as low‐grade PCa (Gleason score <7), and 88 (12.3%) as high‐grade PCa (Gleason score ≥7).

### 2.4 Covariates

A number of variables with potential to confound the associations between BMI and PCa risk were included. These variables were chronological age (years), race/ethnicity (black, white, and other), cigarette smoking (current, former, and never), family history of PCa (yes/no), hypertension (yes/no), benign prostatic disease (yes/no), diabetes (yes/no), chronic kidney disease (yes/no), myocardial infarction (yes/no); medications of aspirin (yes/no), statin (yes/no), insulin (yes/no), finasteride (yes/no); PSA value (ng/mL) and number of PSA testing during the study period.

### 2.5 Statistical analysis

BMI trajectories were detected and quantified utilizing group‐based trajectory modeling method.^22^ This technique permitted objectively grouping of individual participants with similar patterns of BMI change over time. In the analysis, an optimization modeling process was used to determine the number of distinctive trajectories with each trajectory characterized by a linear, binomial, cubic, or quadratic equations. Three criteria were used for optimal model selection: (a) Bayesian Information Criterion (the smaller, the better); (b) all model coefficients characterizing a trajectory were statistically significant at *P *<* *0.05; and (c) the proportion of participants in a detected trajectory group was at least 5% according to the posterior probability.

To associate developmental trajectories with PCa risk, Cox proportional hazards regression modeling was used with age at baseline to the date of PCa diagnosis as time metric. Crude and adjusted hazard ratios (HRs) and 95% confidence intervals (CIs) were estimated to measure the strength of the associations between the BMI measures and PCa risk, overall and by Gleason grade (Gleason score 2‐6 and 7‐10). First, BMI at baseline in categorical and continuous metrics in relation to PCa was analyzed, and linear trends across BMI groups were tested by treating baseline BMI groups from normal weight to extreme obese as an ordinal variable. Then, the association between BMI and PCa risk was assessed by trajectory membership groups determined through the developmental trajectory analysis. Potential confounders, including race, cigarette smoking, family history of PCa, hypertension, benign prostatic disease, diabetes, chronic kidney disease, myocardial infarction, use of aspirin, statin, insulin, finasteride, PSA value (ng/mL) and the number of PSA testing were included as covariates.

The developmental trajectory analysis was conducted using PROC TRAJ, and the Cox proportional hazards regression model was conducted using PROC PHREG. All statistical analyses were conducted using SAS 9.4 (SAS Institute, Carry, NC). *P* values < 0.05 (two‐sided) were reported as statistically significant.

## 3 RESULTS

### 3.1 Baseline characteristics of study sample

Table 1 summarizes the baseline characteristics of the study sample. Among the total 4857 participants, 1428 (29.4%, 1428/4857) were classified as normal weight (18.5 ≤ BMI < 25.0), 1772 (36.5%, 1772/4857) overweight (25.0 ≤ BMI < 30.0), and 1657 (34.1%, 1657/4857) obese (BMI ≥ 30.0); median baseline BMI with interquartile range (IQR) was 27.6 kg/m^2^ (24.4, 31.7). Participants aged 40‐90 years with the median age was 63 (IQR, 54‐72) years. Of the total sample, 714 (14.7%, 714/4857) were diagnosed with PCa, including 626 (87.7%, 626/714) low‐grade (Gleason score <7) and 88 (12.3%, 88/714) high‐grade PCa (Gleason score ≥7).

**Table 1:** Baseline demographic and clinical characteristics of the cohort by categories of baseline BMI

Variables	BMI at baseline (kg/m^2^)			
	<25.0	25.0 to <30.0	30.0 to <35.0	≥35.0
Total sample	1428	1772	998	659
Age, years				
Median	66	64	61	59
Interquartile range	55‐74	55‐72	52‐69	50‐67
Race, n (%)				
White	717 (50.2)	1024 (57.8)	602 (60.3)	387 (58.7)
Black	153 (10.7)	169 (9.5)	113 (11.3)	85 (12.9)
Other	45 (3.2)	50 (2.8)	18 (1.8)	12 (1.8)
Unknown	513 (35.9)	529 (29.9)	265 (26.6)	175 (26.6)
Smoking status, n (%)				
Current smoker	129 (9.0)	102 (5.8)	56 (5.6)	36 (5.5)
Former smoker	357 (25.0)	561 (31.7)	358 (35.9)	224 (34.0)
Never smoke	934 (65.4)	1104 (62.3)	582 (58.3)	399 (60.5)
Unknown	8 (0.6)	5 (0.3)	2 (0.2)	0 (0.0)
Prostate‐specific antigen, n (%)				
<4 ng/mL	665 (46.6)	907 (51.2)	571 (57.2)	381 (57.8)
4‐10 ng/mL	106 (7.4)	92 (5.2)	48 (4.8)	29 (4.4)
>10 ng/mL	36 (2.5)	27 (1.5)	15 (1.5)	12 (1.8)
Unknown	621 (43.5)	746 (42.1)	364 (36.5)	237 (36.0)
Family history of prostate cancer, n (%)	27 (1.7)	27 (1.5)	22 (2.2)	11 (1.7)
Comorbidities, n (%)				
Hypertension	548 (38.4)	799 (45.1)	467 (46.8)	321 (48.7)
Benign prostatic disease	391 (27.4)	470 (26.5)	238 (23.9)	140 (21.2)
Diabetes	181 (12.7)	271 (15.3)	195 (19.5)	179 (27.0)
Chronic kidney disease	114 (8.0)	125 (7.1)	62 (6.2)	48 (7.3)
Myocardial infarction	32 (2.2)	47 (2.7)	22 (2.2)	14 (2.1)
History of medications, n (%)				
Aspirin	281 (19.7)	379 (21.4)	212 (21.2)	137 (20.8)
Statin	280 (19.6)	407 (23.0)	189 (18.9)	130 (19.7)
Insulin	240 (16.8)	308 (17.4)	181 (18.1)	125 (19.0)
Finasteride	43 (3.0)	50 (2.8)	23 (2.3)	15 (2.3)
Follow‐up duration, years				
Median	7.0	8.0	9.0	9.0
Interquartile range	2.0‐12.0	2.0‐13.0	3.0‐14.0	3.0‐14.0

BMI, body mass index.

### 3.2 Conventional analysis of baseline BMI in predicting PCa

We started the analysis with the conventional approach—to associate the baseline BMI as a static measure with incident PCa. The results presented in Table 2 indicate that only overweight at baseline were found to be associated with a marginally higher risk of overall (aHR = 1.22, 95% CI: 1.02, 1.45) and low‐grade PCa (aHR = 1.28, 95% CI: 1.06, 1.54), compared to normal weight at baseline. Although high‐grade PCa accounted for a higher proportion in the groups with baseline BMI of ≥35.0 (19.4%, 13/67) and 30.0 to <35.0 (20.2%, 25/124) than in the groups with baseline BMI of 25.0 to <30.0 (7.9%, 22/280) and <25.0 (11.5%, 28/243), no significant association was found with the obese or overweight groups compared to the normal weight group. These results highlighted the significance in exploring longitudinal BMI dynamics in relation to PCa risk.

**Table 2:** Associations between baseline BMI and PCa risk, results from multivariable Cox proportional hazards regression analysis, overall PCa, and stratified by Gleason grade

	BMI at baseline (kg/m^2^)				*P*_trend_	Continuous, per 5 kg/m^2^
	<25.0	25.0 to <30.0	30.0 to <35.0	≥35.0		
Overall PCa						
No. of cancer patients	243	280	124	67		
Total sample	1428	1772	998	659		
% of cancer patients in total sample	17.0	15.8	12.4	10.2		
cHR [95% CI]	1.00 [reference]	1.07 [0.90,1.28]	1.05 [0.84,1.30]	1.03 [0.79,1.36]	0.708	1.00 [0.96,1.02]
aHR [95% CI]^a^	1.00 [reference]	1.22 [1.02,1.45]	1.24 [0.99,1.54]	1.21 [0.92,1.59]	0.056	1.00 [0.98,1.01]
PCa with Gleason score <7						
No. of cancer patients	215	258	99	54		
Total sample	1400	1750	973	646		
% of cancer patients in total sample	15.4	14.7	10.2	8.4		
% of cancer patients in overall cancer	88.5	92.1	79.8	80.6		
cHR [95% CI]	1.00 [reference]	1.12 [0.94,1.34]	0.98 [0.77,1.24]	1.04 [0.78,1.39]	0.937	0.99 [0.95,1.02]
aHR [95% CI]^a^	1.00 [reference]	1.28 [1.06,1.54]	1.14 [0.90,1.45]	1.24 [0.92,1.65]	0.118	1.00 [0.98,1.02]
PCa with Gleason score ≥7						
No. of cancer patients	28	22	25	13		
Total sample	1213	1514	899	605		
% of cancer patients in total sample	2.3	1.5	2.8	2.1		
% of cancer patients in overall cancer	11.5	7.9	20.2	19.4		
cHR [95% CI]	1.00 [reference]	0.61 [0.33,1.11]	1.40 [0.78,2.51]	0.81 [0.35,1.89]	0.688	0.98 [0.86,1.12]
aHR [95% CI]^a^	1.00 [reference]	0.68 [0.36,1.26]	1.71 [0.94,3.12]	0.95 [0.40,2.24]	0.343	1.00 [0.95,1.05]

aHR, adjusted hazard ratio; BMI, body mass index; cHR, crude hazard ratio; PCa, prostate cancer.

a Adjusted for race, smoking status, prostate‐specific antigen (PSA) level, family history of prostate cancer, histories of hypertension, benign prostatic disease, diabetes, chronic kidney disease, myocardial infarction, medications of aspirin, statin, insulin, finasteride at baseline, and number of PSA testing during study period.

### 3.3 BMI trajectory analysis

**Figure 1:**
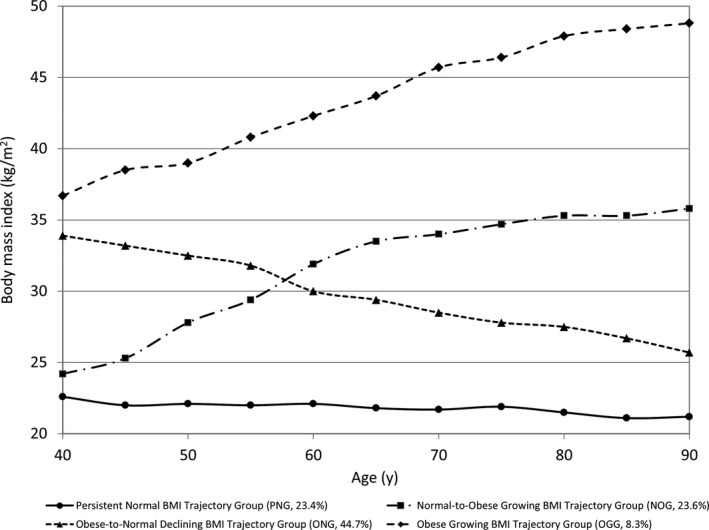
Longitudinal BMI trajectories of men with height and weight measured in clinical settings up to date of prostate cancer diagnosis. Participants were patients seen for prostatic conditions at a tertiary‐care hospital located in Southeastern USA, 1994‐2016

The participants had a median (IQR) follow‐up period of 8.0 (2.0, 13.0) years with a total exposure of 39,078 person‐years. After a series of modeling selection analysis, a four‐group trajectory model (Figure 1) provided the best data‐model fit based on BIC and log likelihood. All model coefficients were statistically significant at *P *<* *0.05 or *P *<* *0.01 level. The group with the smallest proportion contained 8.3% of the total participants, greater than the criterion of 5%. According to the modeling results, the estimated mean BMI by age bellow characterized these four trajectory groups: Group 1: ${\bar{\text{BMI}}}_{40}$ = 22.6, ${\bar{\text{BMI}}}_{50}$ = 22.1, ${\bar{\text{BMI}}}_{60}$ = 22.1, ${\bar{\text{BMI}}}_{70}$ = 21.7, ${\bar{\text{BMI}}}_{80}$ = 21.5, ${\bar{\text{BMI}}}_{90}$ = 21.2; Group 2: ${\bar{\text{BMI}}}_{40}$ = 24.2, ${\bar{\text{BMI}}}_{50}$ = 27.8, ${\bar{\text{BMI}}}_{60}$ = 31.9, ${\bar{\text{BMI}}}_{70}$ = 34.0, ${\bar{\text{BMI}}}_{80}$ = 35.3, ${\bar{\text{BMI}}}_{90}$ = 35.8; Group 3: ${\bar{\text{BMI}}}_{40}$ = 33.9, ${\bar{\text{BMI}}}_{50}$ = 32.5, ${\bar{\text{BMI}}}_{60}$ = 30.0, ${\bar{\text{BMI}}}_{70}$ = 28.5, ${\bar{\text{BMI}}}_{80}$ = 27.5, ${\bar{\text{BMI}}}_{90}$ = 25.7; Group 4: ${\bar{\text{BMI}}}_{40}$ = 36.7, ${\bar{\text{BMI}}}_{50}$ = 39.0, ${\bar{\text{BMI}}}_{60}$ = 42.3, ${\bar{\text{BMI}}}_{70}$ = 45.7, ${\bar{\text{BMI}}}_{80}$ = 47.9, ${\bar{\text{BMI}}}_{90}$ = 48.8. Based on these findings, we termed the four distinctive groups, respectively, as follows: (a) The Persistent Normal BMI Trajectory Group (PNG, 23.4%), (b) the Normal‐to‐Obese Growing BMI Trajectory Group (NOG, 23.6%), (c) the Obese‐to‐Normal Declining BMI Trajectory Group (ONG, 44.7%), and (4) the Obese Growing BMI Trajectory Group (OGG, 8.3%).

### 3.4 Analysis to associate BMI trajectories with PCa risk

Table 3 summarizes the results from Cox proportional hazards regression analysis associating the four BMI trajectories with PCa, overall and stratified by Gleason grade. Compared to those with persistent normal BMI trajectory, participants with normal‐to‐obese growing BMI trajectory had a higher incident PCa risk (aHR = 1.76; 95% CI: 1.25, 2.48) after controlling for covariates. This association was more pronounced for high‐grade PCa (aHR = 2.88; 95% CI: 1.02, 9.05) than for low‐grade PCa (aHR = 1.60; 95% CI: 1.11, 2.30).

**Table 3:** Associations between BMI trajectories and PCa risk, results from multivariable Cox proportional hazards regression analysis, overall PCa, and stratified by Gleason grade

	BMI Trajectory Group			
	Persistent normal BMI Traj.	Normal‐to‐obese growing BMI Traj.	Obese‐to‐normal declining BMI Traj.	Obese growing BMI Traj.
Overall PCa				
No. of cancer patients	193	138	320	63
Total sample	1137	1147	2169	404
% of cancer patients in total sample	17.0	12.0	14.8	15.6
cHR [95% CI]	1.00 [reference]	1.52 [1.07, 2.15]	1.03 [0.87, 1.32]	2.95 [1.27, 6.84]
aHR [95% CI]^a^	1.00 [reference]	1.76 [1.25, 2.48]	1.17 [0.94, 1.47]	3.72 [1.60, 8.66]
PCa with Gleason score <7				
No. of cancer patients	175	116	290	45
Total sample	1119	1125	2139	386
% of cancer patients in total sample	15.6	10.3	13.6	11.7
% of cancer patients in overall cancer	90.7	84.1	90.6	71.4
cHR [95% CI]	1.00 [reference]	1.44 [0.99, 2.08]	1.08 [0.86, 1.35]	2.58 [1.14, 5.82]
aHR [95% CI]^a^	1.00 [reference]	1.60 [1.11, 2.30]	1.19 [0.96, 1.47]	3.46 [1.52, 7.86]
PCa with Gleason score ≥7				
No. of cancer patients	18	22	30	18
Total sample	962	1031	1879	359
% of cancer patients in total sample	1.9	2.1	1.6	5.0
% of cancer patients in overall cancer	9.3	15.9	9.4	28.6
cHR [95% CI]	1.00 [reference]	2.19 [0.69, 6.98]	0.97 [0.47, 2.01]	3.68 [1.25, 8.06]
aHR [95% CI]^a^	1.00 [reference]	2.88 [1.02, 9.05]	0.95 [0.45, 2.20]	4.33 [1.52, 7.74]

aHR, adjusted hazard ratio; BMI, body mass index; cHR, crude hazard ratio; PCa, prostate cancer.

a Adjusted for race, smoking status, prostate‐specific antigen (PSA) level, family history of prostate cancer, histories of hypertension, benign prostatic disease, diabetes, chronic kidney disease, myocardial infarction, medications of aspirin, statin, insulin, finasteride at baseline, and number of PSA testing during study period.

Likewise, the same analysis indicated that compared to persistent normal BMI trajectory, participants with obese growing BMI trajectory were significantly and positively associated with both high‐grade and low‐grade PCa. This association was also more pronounced for high‐grade PCa (aHR = 4.33; 95% CI: 1.52, 7.74) than for low‐grade PCa (aHR = 3.46; 95% CI: 1.52, 7.86). Of note, the risk increase in PCa with the obese growing BMI trajectory was higher than that with normal‐to‐obese growing BMI trajectory.

Lastly, the obese‐to‐normal declining BMI trajectory was not significantly associated with overall or grade‐specific PCa risk, compared to persistent normal BMI, with or without controlling for covariates.

## 4 DISCUSSION

In this study, we successfully conducted a retrospective analysis of longitudinal cohort data derived from electronic medical records from a tertiary‐care hospital with a large sample of 4857 participants. The data allow us to assess a median follow‐up period of more than 8 years with cumulative exposure of a total of 39 078 person‐years to quantify BMI trajectories for a 50‐year age span from 40 to 90 years of age. With this hospital‐based cohort data and derived BMI estimates on an annual basis, we detected and quantified four BMI trajectories. We further statistically associated the BMI trajectories with the risk of newly diagnosed PCa, overall and stratified by Gleason grade. With the rigorous design of retrospectively analysis of longitudinal cohort data and advanced analytical methods, findings of our study add new and scientifically interpretable evidence to the existing literature regarding the relationship between body weight and risk of PCa.

First, the four BMI trajectories detected through the current study are informative to understand body weight change for men since their 40 years of age. The four groups were as follows: (a) PNG (The Persistent Normal BMI Trajectory Group, account for 23.4% of the total sample); (b) NOG (the Normal‐to‐Obese Growing BMI Trajectory Group, account for 23.6% of the total sample); (c) ONG (the Obese‐to‐Normal Declining BMI Trajectory Group, account for 44.7% of the total sample); and (d) OGG (the Obese Growing BMI Trajectory Group, account for 8.3% of the total sample). More than two‐thirds (68.1% = 23.4 + 44.7) of our study participants were categorized into PNG and ONG, suggesting that majority of the men in our sample either maintained or reduced their body weight in the normal range for a long period since 40 years of age. Although all together less than a third (31.9% = 23.6 + 8.3) of total sample in NOG and OGG experienced weight gain during the same period, these men were at increased risk for PCa development.

An important finding in the current study is the association between the four BMI trajectories measured prior to a PCa diagnosis and the incident risk of PCa. According to the adjusted HR, men in the NOG with normal‐to‐obese growing BMI trajectory were 76% more likely to be diagnosed with PCa, and this risk increase was 188% for high‐grade PCa, compared to men in the PNG with persistent normal BMI. In addition, men in OGG with growing obese BMI trajectory were 272% more likely to be diagnosed with PCa and 333% more likely to be diagnosed with high‐grade PCa. Relatively speaking, no impact was observed for men in ONG with obese‐to‐normal declining BMI trajectory. These findings were in line with the results from the Prostate, Lung, Colorectal, and Ovarian Cancer Screening Trial (PLCO), in which risk for high‐grade PCa was increased in men progressing from normal BMI to obesity.^19^

However, our study findings are not in line with some other studies.^12^, ^23^, ^24^, ^25^ Although our study was a retrospective chart review like many others, our observations may have differed because of the approach we took in this study that provided an opportunity to obtain consistent and objective BMI measures over a median span of 8 years of follow‐up. In addition, limitations specified by researchers in these other studies might have contributed to the inconsistence, such as error from self‐reported data for measuring BMI,^24^ and a limited number of time points^12^, ^23^ or a limited time span of BMI measures.^24^, ^25^

Findings in our current study were in line with the general knowledge regarding factors that are associated with body weight and PCa. For example, high‐fat diet and sedentary lifestyle may add to higher PCa risk^26^, ^27^ probably through body weight gain while exercise is protective for PCa^28^ probably through weight control. If age‐related decline in testosterone is risky for the initiation of PCa, weight gain during middle‐to‐late adulthood may alter the testosterone‐estrogen balance due to aromatization of testosterone to estradiol, speeding up testosterone decline.^29^ Recent studies^30^, ^31^ on the dynamic change in testosterone levels provided direct evidence supporting a new mechanism for PCa: quicker‐than‐normal declines in testosterone with age would increase PCa risk. Besides sex hormones, other mechanisms relating weight gain during middle‐to‐late adulthood to PCa risk include insulin, insulin‐like growth factor‐1 (IGF‐1), leptin, and various inflammatory mediators^32^, ^33^; further research at the macropopulation level to understand these mechanisms is needed.

If the findings in our study can be confirmed, it has significant implications for PCa prevention. First, it suggests that maintaining body weight from increasing during middle‐to‐late adulthood can be an effective strategy for PCa prevention regardless if a man is normal weight or overweight. Second, it provides evidence supporting PCa prevention interventions by controlling body weight gain, including low‐calorie diet and physical activity. Lastly, it is worth noting that findings of our study imply that reducing body weight after age 40 years was not associated with reduced PCa risk. Additional studies are needed to confirm this result.

Strengths in the current study include that the data for this analysis were extracted from electronic health records, thereby avoiding the probability of recall bias. With 39,078 person‐years and a median of 8.0 years of follow‐up, we were able to identify a significant number of PCa patients, thus this study was well powered to investigate the outcome of interest. Besides, we derived BMI data on an annual basis with a minimum of three BMI measurements at different years, improving our ability to better detect the longitudinal dynamics of BMI change. In addition, linking the databases of demographic information, laboratory results, drug prescriptions, and diagnoses allowed us to collect and adjust for a number of confounders, including race, smoking status, PSA levels, and cumulative number of PSA testing.

There are several limitations in our study. First, noncancerous participants were selected from those also with a history of urologic clinic visit but due to a benign prostatic disease and/or PSA >4 ng/mL. This increased the possibility for men with higher BMI to be included, because obesity was also associated with benign prostatic diseases.^34^ Although this bias might have diluted the estimated association between BMI trajectory and PCa risk, caution is still needed when generalizing findings from our study to other populations. Second, the height and weight data for BMI assessment in our study, although were directly measured by healthcare providers rather than from participants' self‐report, were not collected on predetermined time intervals. Therefore, the detected BMI trajectories may be subject to some errors because of variations in BMI measurement frequency and intervals for different study participants. Third, the current study sample only included those with repeatedly measured height and weight before PCa diagnosis or censoring, we cannot rule out the possibility that this sample was not random, particularly considering only 12.3% (88/714) of the PCa patients were of Gleason score ≥7. More studies are needed to replicate the findings in the current study. Additionally, we tried to replicate the analysis among those with follow‐up >5 years (n = 3272), among which, however, only 192 (5.9%, 192/3272) were PCa patients, the remaining 3080 (94.1%, 3080/3272) were non‐PCa. Among the 192 PCa, 155 (80.7%, 155/192) were low‐grade and 37 (19.3%, 37/192) were high‐grade. We admit that the relationship between BMI trajectory and cancer grade among those with >5 years of follow‐up will be more valid to reach a conclusion, but due to the small sample size of PCa patients with >5 years of follow‐up, we expect the analysis power to be low, and hope for an opportunity to explore this in the future.

Despite these limitations, the current study, using electronic medical records data ranging from middle to late adulthood and BMI trajectory modeling, demonstrated that a growing BMI trajectory is associated with increased PCa risk, especially for high‐grade PCa, through middle‐to‐late adulthood. Further studies are warranted to confirm this finding.

## CONFLICT OF INTEREST

None declared.
