# Supplementary material for: BMI trajectories and risk of overall and grade‐specific prostate cancer: An observational cohort study among men seen for prostatic conditions
Source: Cancer Med. 2018 Sep 11;7(10):5272–80. doi: 10.1002/cam4.1747 (PMC6198207; doi:10.1002/cam4.1747)
Supplement: Supplementary file 1 [file CAM4-7-5272-s001.docx]

**Supplementary Material**

BMI Trajectories and Risk of Overall and Grade-Specific Prostate Cancer: An Observational Cohort Study among Men Seen for Prostatic Conditions

**Table of contents**

**Supplementary Table s1:** ICD-9 codes of prostatic conditions used in this study.

Supplementary Table s1. ICD-9 codes of prostatic conditions used in this study.

| ICD-9 code | ICD-9 code description |
| --- | --- |
| 98.12 | Gonococcal prostatitis (acute) |
| 98.32 | Gonococcal prostatitis chronic |
| 131.03 | Trichomonal prostatitis |
| 185 | Malignant neoplasm of prostate |
| 222.2 | Benign neoplasm of prostate |
| 233.4 | Carcinoma in situ of prostate |
| 236.5 | Neoplasm of uncertain behavior of prostate |
| 600 | Hypertrophy (benign) of prostate without urinary obstruction and other lower urinary tract (luts) |
| 600.01 | Hypertrophy (benign) of prostate with urinary obstruction and other lower urinary tract symptoms (luts) |
| 600.1 | Nodular prostate without urinary obstruction |
| 600.11 | Nodular prostate with urinary obstruction |
| 600.2 | Benign localized hyperplasia of prostate without urinary obstruction and other lower urinary tract symptoms (luts) |
| 600.21 | Benign localized hyperplasia of prostate with urinary obstruction and other lower urinary tract symptoms (luts) |
| 600.3 | Cyst of prostate |
| 600.9 | Hyperplasia of prostate, unspecified, without urinary obstruction and other lower urinary symptoms (luts) |
| 600.91 | Hyperplasia of prostate, unspecified, with urinary obstruction and other lower urinary symptoms (luts) |
| 601 | Acute prostatitis |
| 601.1 | Chronic prostatitis |
| 601.2 | Abscess of prostate |
| 601.3 | Prostatocystitis |
| 601.4 | Prostatitis in diseases classified elsewhere |
| 601.8 | Other specified inflammatory diseases of prostate |
| 601.9 | Prostatitis unspecified |
| 602 | Calculus of prostate |
| 602.1 | Congestion or hemorrhage of prostate |
| 602.2 | Atrophy of prostate |
| 602.3 | Dysplasia of prostate |
| 602.8 | Other specified disorders of prostate |
| 602.9 | Unspecified disorder of prostate |
| 790.93 | Elevated prostate specific antigen (PSA) |
